# Interatrial Block: A Virtual Pandemic Requiring Attention

**Published:** 2014-03

**Authors:** Raman Mehrzad, David H. Spodick

**Affiliations:** 1 Department of Medicine, Steward Carney Hospital, Boston, MA, USA;; 2 Department of Medicine, Division of Cardiology, St. Vincent Hospital and University of Massachusetts Medical School, Worcester, MA, USA

**Keywords:** Electrocardiography, Pandemic, Atrium, Block

## Abstract

Interatrial block (IAB) denotes a conduction delay between the two atria (P-wave duration ≥110 ms). Depending on the severity of the block, IAB can be partial or advanced. Even though several studies have reported a high prevalence of IAB, it still remains a diagnosis many neglect without any follow-up. The crisis in IAB is undramatic until predictable complications appear. Nevertheless, the danger in IAB is real because of the major associations with multiple medical conditions, including atrial fibrillation, myocardial ischemia, left atrial enlargement, and systemic emboli. There are different treatment options for IAB to eliminate its consequences, including pacing and medical management with angiotensin-converting enzyme inhibitors and angiotensin receptor blockers. Pacing has been shown to give promising results and could potentially prevent conditions related to cardiovascular disease such as hypertension or diabetes mellitus. Given the high prevalence of IAB, together with its potentially serious consequences, and yet being largely ignored, we stress attention to this potentially dangerous pandemic and raise consideration for further investigations.

## Introduction

Interatrial block (IAB) signifies a conduction delay between the two atria. Impaired conduction of an impulse through the atria is manifested by a widened and often notched P-wave. A P-wave duration≥110 ms is the diagnostic criterion.^1^^-^^3^ Although present in remarkably high prevalence  and well described in the literature, this cardiac conduction disorder is poorly recognized. Nevertheless, its major association with atrial fibrillation and other supraventricular tachycardias, peripheral embolism,^4^^-^^6^ and impaired left atrial electromechanical function emphasizes its clinical and epidemiologic significance. Yet, many textbooks in general medicine^7^^-^^9^ and even cardiology^10^^-^^12^ do not mention, nor discuss IAB and its associations with other clinical conditions. Also, many articles fail to report its true prevalence by restricting investigation either to lead II alone or cite only one or two other leads.^3^ We did a comprehensive literature review, using PubMed as our main source and also book chapters that cover this topic, and summarized and concluded the results. Our overall selection of articles was peer-reviewed articles and book chapters with a relevant focus on the definition, prevalence, pathophysiology, outcome, and therapeutic strategies of IAB. Our purpose is to call attention to this situation by defining IAB, stress the results of analysis, report its prevalence and association with other conditions, and finally review more recent results of investigation.

## Definition of Interatrial Block

**Figure 1:**
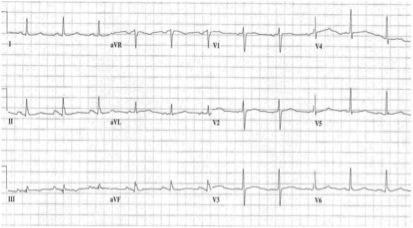
Electrocardiogram with interatrial block

Normal P-wave duration is considered ≤100 ms, representing the normal transit time for electrical impulses generated in the sinus node to be conducted throughout the right and left atria (RA and LA). IAB is defined as prolonged interatrial conduction, with P-wave prolongation ≥110 ms, due to impulse slowing or blockage, frequently, but not exclusively, in the Bachmann bundle (BB), prolonging P-wave duration (figure 1). First-degree IAB is when P-wave duration is >110ms. In third-degree IAB, there are longer P-waves with biphasic morphology in the inferior leads. Lastly, in second-degree IAB, these patterns appear transiently in the same ECG recording (atrial aberrancy).^13^ Problems when interpreting prolonged P-waves are well known; it might be caused by either reduced conduction velocity via interatrial connections, atrial enlargement, elevated atrial filling pressure, and other factors, which is why it is difficult to delineate the exact cause of P-wave prolongation. For this reason, the most recent guidelines review the complexity of the definition and, due to our inability to exactly identify the cause of P-wave abnormality, recommend the continued use of the term: “left atrial abnormality”.^14^

To improve specificity, investigators used P-wave durations ≥120 ms; others used P-wave durations ≥130 ms in signal-averaged ECGs for the detection of filtered P-wave duration.^15^ It was later found that the maximum duration of P waves in IAB was most often in leads II, aVF, and V_5_, but the widest P-wave (defining the degree of block) could be found in any lead. Most physicians and many medical textbooks continue to define the P-wave abnormality of IAB as “LA abnormalty”,^14^ which might be associated with left atrial enlargement (LAE) seen, before modern imaging, in patients with IAB during autopsies. However, although likely to be present in patients with IAB, LAE is not the precise interpretation; the ECG shows a prolonged P duration.

## Prevalence of Interatrial Block

Previous studies showed a high prevalence of IAB. In two unrelated general hospital populations, IAB was discovered in more than 40% of patients with sinus rhythm and 60% of patients older than 59 years. In the healthy pediatric population, however, recent studies have shown that abnormal P-wave morphology, signifying the presence of IAB, is very rare.^16^ In young healthy men, IAB was present in only 9% of those younger than 35 years and 5% of those younger than 20 years.^17^

The measurement of P-wave duration is usually done by using a single lead (lead II) or a combination of only 2 or 3 leads. However, more recent studies have specified the diagnostics by using all 12 leads to determine maximum P-wave duration.^1^^,^^2^^,^^17^

P-wave indices were shown, by the Framingham Heart Study, to correlate with advancing age. Longer mean P-wave indices were seen in healthy patients without cardiovascular disease, hypertension, diabetes, or obesity, and in elderly patients.^18^

## Partial Versus Advanced Interatrial Blcok

**Figure 2:**
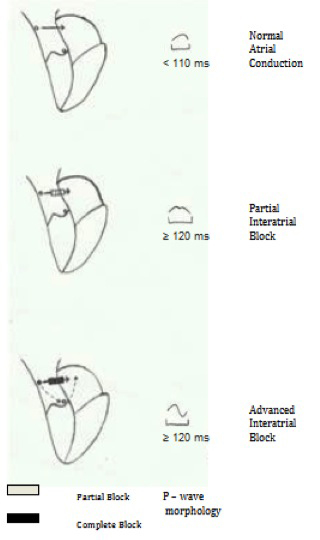
Partial and advanced block

Depending on the severity of the block, IAB can be partial or advanced. Wide, usually bifid P waves are produced in a partial block, which is seen when impulses travel from the RA to the LA via the BB or other routes when interatrial conduction is delayed.^2^^,^^19^ Thus, when interatrial conduction is normal, the impulse leaves the sinus node and crosses from the RA to the LA in the BB and other connections. Most commonly, we find partial IAB, where the impulse still crosses as it does with normal interatrial conduction, but is delayed, and P-wave duration is >100 ms (figure 2). When conduction is completely blocked (advanced IAB), sinus impulses cannot cross to the left but must travel inferiorly in the RA toward the atrioventricular junction and thereafter superiorly through the LA.^19^

The impulse is completely blocked for its usual manner of crossing from the RA to the LA, but descends in the RA to the area of the atrioventricular node, after which it activates the LA in reverse. Thus, in advanced IAB, the RA is activated in an orthograde direction and the LA is activated in a retrograde direction.^3^

Partial IAB can progress to advanced IAB. Progression time from partial IAB to advanced IAB is shorter than that of the normal P-wave to advanced IAB.^20^ As was previously thought, advanced IAB may not exclusively be a complete block.^21^

## Risk Factors and Pathophysiology of Interatrial Block

Although the exact pathophysiology of impaired interatrial conduction remains largely unknown, some studies have shown intracellular fibrotic changes and metabolic inclusions in tissue from patients with IAB, particularly in the sarcomere and sarcoplasmic reticulum.^22^ Generally, coronary artery disease, which contributes to atherosclerotic plaque formation and endothelial injury, might lead to ischemia-mediated interatrial conduction delay. Thus, cardiovascular risk factors such as diabetes mellitus, hypercholesterolemia, hypertension, obesity, smoking, physical inactivity, and increasing age have been identified as risk factors for developing IAB.^23^  There are also studies that have supported this by showing a significant reduction in P-wave duration after angioplasty in patients with acute myocardial infarction.^24^ Progressive systemic sclerosis and possibly other autoimmune disorders may also impair arterial circulation, including in the BB, and lead to the development of IAB.^25^ Moreover, amyloidosis, lymphoma, and hypertrophic cardiomyopathy involving the atrial septum, especially its superior portion near the BB, can produce similar interatrial conduction delay (table 1).^26^^,^^27^

**Table 1:** Risk factors and pathophysiology of interatrial block

**Potential risk factors **	**Possible pathophysiology**
Coronary artery disease	Intracellular fibrotic changes
Cardiovascular risk factors	Metabolic inclusions
Autoimmune disorders	Ischemia
Amyloidosis	
Lymphoma	
Cardiomyopathy	
Congestive heart failure	
Valvular disorders	
Hypervolemia	Atrial stretch

Increased atrial filling pressure and overstretch of the atrium in conditions such as congestive heart failure, valvular disorders, and hypervolemia may also cause prolonged conduction or unmask already slowed impulse transmission in the interatrial conduction pathways. Since diuretic therapy for these can reduce P-wave duration, this statement is further supported.^28^

## Potential Outcomes of Interatrial Block

*Interatrial Block and Left Atrial Size*

There are a number of significant concerns in patients with IAB. Patients with IAB tend to have increased LA volumes and diameters. These patients have longer left ventricular Doppler A-wave acceleration times and significantly lower LA stroke volumes, LA ejection fractions, and LA kinetic energy (table 2).^29^^,^^30^ Thus, IAB results in both delayed LA activation and delayed atrial contraction and potentially sets the stage for mistimed LA contraction against a closed or closing mitral valve, which results in a rise in LA pressure, increasing LA wall stress, and subsequent LA dilatation.^29^^,^^31^ IAB patients were matched with those who had normal LA and with a control series that included patients with enlarged LA without IAB. This demonstrated that IAB is associated with a sluggish, poorly contractile LA, and the degree of dysfunction is directly related to the degree of conduction delay between the RA and the LA (represented by maximum P-wave duration). The relation between P-wave duration and left atrial size in terms of long-axis diameter was: LA dimension (millimeters)=2.47+0.29 (P-wave duration in milliseconds). In one study, the ECG was compared with cardiovascular magnetic resonance criteria for LAE and demonstrated that the prevalence of LAE by using the ECG criteria of P-wave duration >110 milliseconds was 70%. However, by using cardiovascular magnetic resonance criteria, the prevalence of LAE was found to be only 28%.^32^ P-wave duration >110 milliseconds was sensitive (84%) but lacked specificity (35%) in the detection of LAE. This confirms that although IAB is commonly found with LAE, it can also occur independently of increased atrial size. On the other hand, enlarged atria certainly should require longer total activation time and thus directly affect the morphology of the P wave. Thus, P-wave morphology is indeed a complex outcome of anatomic and electrophysiological factors, both affecting the way the sinus impulse travels across the atria.

**Table 2:** Left atrium parameters and P-wave duration in patients with interatrial block

**Variables**	**Control subjects**	**Patients with interatrial block**	**P value**
P-wave duration, ms	96.0±15.0	141.0±17.0	<0.0001
P-wave amplitude, mV	0.11±0.002	0.11±0.005	0.71
Left atrial diameter, mm	44.1±3.3	45.1±5.5	0.52
Maximal left atrial volume, mL	91.4±42.3	107.1±33.8	0.20
Minimal left atrial volume, mL	58.6±29.0	85.5±30.7	0.008
Left atrial volume at onset of atrial systole, mL	75.8±33.0	92.7±31.3	0.11
Left atrial systolic velocity, mL	17.3±6.0	7.2±5.0	<0.0001
Left atrial ejection fraction, %	24.6±709	8.5±6.1	<0.0001
Left atrial kinetic energy, kdyne/cm/s	64.7±4	19.8±14	<0.0001
Acceleration time, ms	83.1±24.0	114.7±39.0	0.007

*Interatrial Block and Left Atrial Function*

Since most patients with IAB have a large and poorly contracting LA with reduced and delayed left ventricular (LV) filling, IAB is associated with LA electromechanical dysfunction (table 2). In a series of patients matched for LA size, those with IAB had lower LA emptying fraction, lower LA stroke volume, and lower LA kinetic energy.^29^ With a weak and enlarged LA, this could intensify the risk for thrombosis and subsequent arterial embolism. It has been demonstrated that patients with embolic stroke had 80% prevalence of IAB, which is twice that of the index population.^33^ A following cohort study in patients with embolic stroke also highlighted an exceptionally high prevalence of IAB.^34^

P-terminal force (Ptf) may indicate LA abnormality, particularly LA enlargement. There is a significant correlation between IAB and P-terminal force.^35^ Remarkably, IAB was found in 62% of patients who had Ptf and, therefore, ECG interpreters should be encouraged to search for IAB when P-wave negative terminal force is identified.^35^

Signal-averaged P-wave and orthogonal P-wave analysis are the other noninvasive ways of assessing interatrial conduction. In addition, the P-wave morphologies derived from these methods have been shown to correlate with the interatrial routes used.^35^^-^^37^

Lastly, there are studies suggesting a molecular and pathophysiological relationship between diastolic dysfunction and the electromechanical remodeling of the LA; however, it is not definite which is first and which is last, which implies the existence of a vicious cycle.^38^

*Interatrial Block and Arrhythmias*

Several studies have identified correlations between IAB and atrial arrhythmias, particularly atrial fibrillation (AF).^39^ In one study, the prevalence of IAB in patients with paroxysmal AF was 52%. The large sluggish LA of IAB suggests that, with the onset of AF, stasis and ultimately LA and LA appendage thrombosis are likely. This is the basis for the well-known association between untreated AF and peripheral arterial emboli, particularly cerebral emboli. Because early AF tends to be paroxysmal, such an event may be the first evidence of arrhythmia or IAB. Moreover, the risk for developing atrial arrhythmia is also substantially higher in patients with advanced IAB.^6^ Furthermore, the onset and offset of paroxysmal arrhythmias are associated with a higher tendency for embolization, indicating that atrial thrombosis would have preceded them. Furthermore, p-wave analysis, including p-wave dispersion, and IAB can predict AF.^16^

Prolonged atrial conduction is also a predisposing factor for the development of atrial flutter, where the mechanism for atrial arrhythmias is mainly due to the abnormal impulse conduction between the atria along interatrial pathways, primarily the Bachmann’s Bundle, where atrial conduction times are increased.^40^^-^^44^

*Interatrial Block and Left Ventricular Function*

With respect to LV function, IAB can give >30 ms mean delay in active (atriogenic) LV filling, associated with a considerably late activation of the LA.^45^ The compromised atrial “kick” from a sluggish LA and, particularly, the greatly reduced LA stroke volume and LA kinetic energy produce significantly reduced preload, additionally suggesting increased risk for congestive heart failure in patients with IAB.^29^ Two recent studies have demonstrated that hemodynamic evolution of acute decompensated heart failure patients could be accessed by ECG analysis, specifically P-wave duration, although this was only seen in one case.^46^^-^^48^ Moreover, these studies have also shown how well P-wave morphology and duration correlate with the clinical course, development, and serum level of B-type natriuretic peptide.^47^^,^^48^

*Interatrial Block and Ischemia*

IAB has been described as an additional predictive marker in detecting ischemic heart disease.^49^ Several studies have identified a significant relation between P-wave duration and ischemia during exercise tolerance tests.^50^^-^^52^ It has been shown that when a P-wave duration ≥120 milliseconds during exercise stress tests was added to the conventional criteria for diagnosing ischemia, sensitivity would increase from 57% to 75% while specificity would drop only from 85% to 77%.^51^ Also, there was a greater incidence of IAB during exercise in patients with evidence of myocardial ischemia, in comparison to those without. Furthermore, the Duke Prognostic Treadmill Score, shown in a recent study, is indeed inversely associated with P-wave duration and was more significant with P-wave increases >20 milliseconds than with P-wave increases ≤20 milliseconds.^52^ P-wave duration or IAB is, thus, a promising factor in facilitating the diagnosis of myocardial ischemia. However, larger randomized controlled studies are required to verify this.

A recent study with 172 patients with acute occlusive mesenteric ischemia showed that the prevalence of IAB was 88.9%, which demonstrates that  IAB can be a novel risk factor for acute mesenteric ischemia.^53^

*Interatrial Block Association with Other Diseases*

Very few investigations have been done to show associations between IAB and disease states that potentially affect P-wave morphology. However, recent studies have demonstrated how P-wave duration could reflect the evolution of acute heart failure and its association with the clinical course.^47^^,^^48^ Moreover, it was demonstrated that a severely increased mitral gradient, mitral valve annuloplasty, increased pulmonary artery pressure, and poor New York Heart Association (NYHA) class correlated with IAB duration and P-terminal force. Significant IAB (>or=120 ms) and P-terminal force might be considered as a novel correlate of echocardiographic severity and associated complications during the follow-up of mitral stenosis.^54^ Furthermore, P-wave prolongation in patients with hyperthyroidism has been reported.^15^ However, further investigations are needed to explore these results and other possible diseases that might be associated with P-wave morphology and diagnosis of IAB.

Early recognition of IAB could also potentially allow the identification of existing diseases, like asymptomatic Friedreich’s ataxia patients, who are prone to develop potentially life-threatening arrhythmias.^55^ Furthermore, moderate to severe obstructive sleep apnea are predictors of IAB; P-wave dispersion is generally increased in these patients, which might explain the high prevalence of atrial arrhythmias.^56^

## Therapeutic Strategies for Interatrial Block

IAB can be corrected using biatrial pacing, dual-site RA pacing, single-site interatrial septal pacing, or BB pacing.^57^^-^^59^ Furthermore, by improving LV function and reducing LV end-diastolic pressure and LA filling pressure, cardiac resynchronization therapy can also reduce P-wave duration. However, further investigations are needed to determine the optimal pacing approach.

There are also medical options for the treatment of IAB. Angiotensin-converting enzyme inhibitors (ACEi) and angiotensin receptor blockers (ARBs) can, as shown in several studies, control and prevent AF.^60^^-^^62^ ACEi and ARBs can significantly reduce P-wave duration in these patients.^62^^,^^63^ This is also the case in patients with hypertension, where ACEi and ARBs substantially decrease P-wave duration.^63^^,^^64^ Thus, treatment with ACEi and ARBs can theoretically slow the progression of IAB, possibly via suppression of atrial fibrosis by cytokine modulation and cardiac remodeling, or through unloading pressure- and stretch-overloaded atria.^60^^,^^65^ ACEi, or a combination of an ACEi and *β*-adrenergic blocker can also significantly delay the progression time in patients who have progressed from partial to advanced IAB, as was shown in a recent study.^23^ One case study also showed the resolution of advanced IAB to partial IAB during exercise, following the administration of a *β*-adrenergic blocker. However, this issue is still debated and in contrast to the above findings, there are randomized controlled studies such as the CAPRAF-study that show neutral results.^66^

Anticoagulation has been proposed as treatment in patients with IAB to prevent embolic stroke. However, prospective controlled trials with a large sample size are needed.^67^

## Conclusion

IAB has a largely overlooked pandemic incidence in hospitals, both in- and out-patient settings, with numerous and remarkably significant elements. While identifying IAB is not difficult or complicated as compared to other ECG abnormalities, it is largely unrecognized, even with reading by ECG computers. 

As was demonstrated above, the prevalence of IAB is remarkably high in general hospital populations. Many institutions and investigators, therefore, use P-wave durations ≥120 milliseconds to identify IAB. Is the standard criterion for ECG diagnosis of IAB (P-wave duration ≥110 milliseconds) inaccurately defined? Since the prevalence of IAB is high, and the standard criterion for ECG diagnosis differs among investigators, it is reasonable for the criterion for IAB to be re-evaluated to set a clinically relevant standard. However, this has to be established after studies show that a potential increase in the threshold can make a significant difference for further management, with confirmed specificity and sensitivity. Regardless, the standard criterion for ECG diagnosis should be set to a level of relevance where clinicians acknowledge the diagnosis and follow-up for further investigation. The emphasis on work-up in these patients can potentially prevent future cardiovascular outcomes.

Similar to determining other important ECG criteria with modern epidemiologic studies, epidemiologic data for IAB should also be made available and taken into consideration, evaluating the full 12-lead ECG to detect the true maximum P-wave duration and P-wave morphology, which increases sensitivity with the number of leads used. Consequently, the importance of multitrials detecting the cut-off values of IAB and risk should be underscored. 

Although the clinical consequences of IAB may be grave, absence of sufficient epidermiologic investigations and controlled trials means that no guidelines can be constructed for managing IAB patients. Do these patients need immediate treatment (i.e., ACEI, anticoagulation, and/or antiarrhythmic therapy?) If so, should it be prophylactic, anticipating atrial arrhythmias, i.e., anticoagulation? While such investigations are needed, electrophysiologic studies are inconvenient, costly, and unsuitable as a screening tool among the general population. Clinically, the ECG is an excellent diagnostic tool for demonstrating abnormal interatrial conduction. Therefore, ECGs should be carefully analyzed by clinicians by reading all leads and not only focus on one (usually lead II) or a few leads, to better detect IAB and cultivate an awareness of its potentially dangerous prevalence and consequences. 

We stress attention to this potentially dangerous pandemic and raise consideration for further investigations. In addition, further research is needed to reveal more data on the definitive role of IAB and the optimal management to preclude its consequences.
